# Single level cervical disc herniation: A questionnaire based study on current surgical practices

**DOI:** 10.4103/0019-5413.53453

**Published:** 2009

**Authors:** Saeid Abrishamkar, Yousef Karimi, Mohammadreza Safavi, Pouria Tavakoli

**Affiliations:** Department of Neurosurgery and Intensive Care unit, Isfahan University of Medical Sciences, Isfahan, Iran; * Department of Anesthesiology and Intensive Care unit, Isfahan University of Medical Sciences, Isfahan, Iran

**Keywords:** Anterior approach, cervical disc herniation, discectomy without fusion, disectomy with fusion

## Abstract

**Background:**

Operative procedures like simple discectomy, with or without fusion and with or without instrumentation, for single level cervical disc herniation causing neck pain or neurological compromise have been described and are largely successful. However, there is a debate on definitive criteria to perform fusion (with or without instrumentation) for single level cervical disc herniation. Hence, we conducted a questionnaire based study to elicit the opinions of practicing neurosurgeons.

**Materials and Methods:**

About 148 neurosurgeons with atleast 12 years of operative experience on single level cervical disc herniation, utilizing the anterior approach, were enrolled in our study. All participating neurosurgeons were asked to complete a practice based questionnaire. The responses of 120 neurosurgeons were analysed.

**Results:**

The mean age of enrolled surgeons was 51 yrs (range 45-73) with mean surgical experience of 16.9 yrs (range 12-40 yrs) on single level cervical disc herniation. Out of 120 surgeons 10(8%) had 15-25 years experience and always preferred fusion with or without instrumentation and six (five per cent with 17-27 yrs experience had never used fusion techniques. However, 104 (87%) surgeons with 12-40 yrs experience had their own criteria based on their experiences for performing fusion with graft and instrumentation (FGI), while. 85 (75%) preferred auto graft with cage.

**Conclusions:**

Most of surgeons performed FGI before the age of 40, but for others, patient criteria such as job (heavier job), physical examination (especially myelopathy) and imaging findings (mild degenerative changes on X-ray and signal change in the spinal cord on MRI) were considered significant for performing FGI.

## INTRODUCTION

Single level cervical disc herniation causing neck pain and / or radicular pain with or without neurological compromise is a common affliction. The surgical treatment options which include simple discectomy with or without fusion and with or without instrumentation are largely successful in yielding excellent relief of signs and symptoms post operatively and during followup.^1^ Although each procedure has its merits and demerits, it is still unclear if one surgical technique is superior over the other.^1^ Operative time and hospital stay were found to be slightly shorter for anterior cervical discectomy compared with anterior cervical discectomy with fusion.^1^ The clinical and radiographic findings of some of the common techniques of anterior fusion for cervical disc herniation such as Cloward fusion with autograft, Cloward fusion with freeze-dried bone, BAK-C device fusion and cage have the advantage of affording more stability, with less chances of development, of late kyphotic deformity, but on the other hand, there is concern related to graft donor site morbidity, and / or devices and the need for more manipulative force needed intra operatively for graft or device insertion during operation.^2^ Although during the last three decades, the anterior approach to the cervical spine with inter-body fusion has been increasingly preferred, but some other retrospective studies on surgical procedure without interbody fusion report that the bone graft and hence fusion is not important for successful operative treatment of single level herniated cervical disc.^3^

However, none of the operative procedures are considered to be superior over the others and many surgeons believe or suggest that the selection of surgical procedure may reasonably be based on the preference of the surgeon tailored to the individual patient.^1^ Those neurosurgeons who believe in fusion with instrumentation claim that this technique has significant advantages, including higher patient satisfaction, unnecessary postoperative collar, early rehabilitation and lower rate of complications but others do not believe so.^2^ In some studies an excellent or good long-term result was achieved in 90.9% of the patients with single level disc herniation with radiculopathy and 58.1% of those with myelopathy operated with anterior discectomy without fusion.^2^ The age of the patients, the duration of symptoms before diagnosis and pathogenesis of disc herniation did not represent significant parameters influencing the outcome of patients.^1^,^2^

Hence a prospective questionnaire based study was designed to evaluate the opinion of participating neurosurgeons with wide experience on operative treatment of single level cervical disc herniation to decipher their decision making criteria on their preferred operative procedure.

## MATERIALS AND METHODS

In the prospective study 148 neurosurgeons performing surgery for single level disc herniation with anterior approach for atleast last 12 years and performing more than 20 operations each year were enrolled. All of the participating neurosurgeons were asked to complete a pre-structured questionnaire according to their clinical preferences. The 28 neurosurgeons who were performing less than 20 operations each year were excluded, leaving 120 questionnairres for the final analysis.

The surgeons were asked if they preferred fusion for single level cervical disc herniation with anterior approach; and whether they preferred to perform discectomy with or without fusion and / or instrumentation. The anterior techniques offered that were asked were the following; discectomy without fusion, discectomy with autograft fusion without instrumentation, discectomy with autograft and Plaque Screw Devices (PSD) and discectomy with cage (with autograft or hydroxyapitate).

The questionnaire was designed to know the decision making criteria utilized by surgeons for their particular operative procedure using the anterior approach to single level cervical disc herniation. The following parameters, based on various patient demographics, clinical findings and imaging studies were asked, such as age, job, physical findings on clinical examination, image finding including; plain X-ray and MRI of the cervical spine and finally any previous operation to which the patient might have been subjected.

The patients were divided into three groups; less than 40, 40 to 70 and over 70 years. Any effect of sex of the patient which may influence the type of operative procedure was asked. To study any possible effect of job on decision making, it was divided into heavy or ordinary job. Heavy job / occupation was defined as the one which required physical activity or a mean resultant force over the neck.

The common physical examination findings suggestive of degenerative disc disease of the cervical spine may include neck pain, radiculopathy, and myelopathy. These symptoms may occur alone or in combination. All these were a part of the questionnaire. The findings on X-ray and MRI of the cervical spine suggestive of degenerative changes such as sclerosis of adjacent vertebral body or osteophyte formation and the manner in which that may affect the operative decision making were asked. The questionnaire is shown on Table 1. Those who did not answer the complete questionnaire were excluded. All information of the check list was analyzed with SPSS software.

**Table 1:** Questionnaire to evaluate criteria of neurosurgeons on single level cervical disc herniation with anterior approach

What is your preferred strategy in single level cervical disc herniation? I never do fusion. I always do fusion. I sometimes do fusion. (If your answer is 2 or 3, please answer the rest of questionnaire) What is your preferred technique in single level cervical disc herniation? Discectomy and autograft bone without instrument. Discectomy and autograft bone with plaque and screw. Discectomy and fusion with ceramics (hydroxyapatite), plaque, Screw. Discectomy, fusion with autograft bone or hydroxyapatite and cage. Which one of mentioned methods did you use previously? Since how many years have you used the new technique? Please choice the following item according to the way they have influence on your decision making: Age: I use my prefer technique in patients before age forty. I use my prefer technique in patients between age forty to seventy. I use my prefer technique in patients after age seventy. Age has no effect on my decision. Sex: I use my prefer technique in men. I use my prefer technique in women. Sex has no effect on my decision. Job: I use my preferred technique in patients with heavier occupations. I use my preferred technique in patients in lighter occupations. Occupations have no effect on my decision. Clinical findings: I use my preferred technique when patient has radiculopathy. I use my preferred technique when patient has myelopathy. I use my preferred technique when patient has cervical pain. Clinical findings have no effect on my decision. X-ray findings: I use my preferred technique when patient has severe degenerative changes in x-ray. I use my preferred technique when patient has mild degenerative changes in x-ray. X-ray findings have no effect on my decision. MRI findings: I use my preferred technique when patient has degenerative signal changes at the same level of spinal cord. MRI findings have no effect on my decision. Previous operation in same level: I always use fusion in this patient. I never use fusion in this patient. Previous operation has no effect on my decision.

## RESULTS

All 120 respondants who completed the questionnaire were subjected to data analysis. The mean age of participating neurosurgeons was 51 (range 45- 73 years) and had an average operative experience of 16.9 years (range 12-40 yrs) on single level cervical disc herniation. Of them, 104 surgeons (87%) sometimes used fusion with graft and instrumentation (FGI); 10 (8%) surgeons always used fusion with or without instrumentation whereas 6 (5%) never used fusion techniques.

76 surgeons had changed their surgical technique over the last 5.8 years from Cloward's procedure (n=4), autograft bone without instrument (n=52) and autograft bone with plaque and screw (n=20) to FGI. Of the 114 (95%) surgeons who always or sometimes prefer fusion for single level cervical disc herniation, one preferred ceramics, six preferred only autograft without instrumentation (Smith and Robinson or Cloward technique), 22 preferred FGI (autograft with plaque and screw), and 85 (75%) preferred FGI with cage.

Out of 120 surgeons, for 102 (89%) age of the patient was the most important decision making criteria and in this group 100 (98%) preferred fusion in patients before age 40 and none after the age of 70. Patient's occupation was the second most important criteria considered. While 98 (86%) surgeons preferred FGI in patients with heavy job, 64 (53%) surgeons believed that in the presence of myelopathy it is better to consider FGI, but for 14 (12%) surgeons radiculopathy and neck pain were also a significant criterion to consider FGI whereas two surgeons didn't have any opinion on this issue.

Sevety two surgeons believed that in the presence of mild degenerative change in the cervical spine it is better to consider FGI, whereas, 28 neurosurgeons did not think so and 14 had no opinion on this criterion. The other six neurosurgeons did not answer to this criteria. Signal changes on MRI, were considered to be important by 62 surgeons and 48 preferred FGI in the presence of mild to moderate signal change in spine and disc space. When signal change was detected in the spinal cord on MRI including hypersignal gliosis on T1 and T2 weighted, 51 surgeons preferred the technique of FGI;. 18 surgeons didn't answer this query or this criterion was not important for them. 72 surgeons had done FGI if any previous operation was done earlier at the same vertebral or disc level the rest didn't have any idea or experience with such a situation. Sex of the patient had no bearing on decision making for any of the respondants.

## DISCUSSION

The controversy regarding ideal surgical procedure for single level cervical disc herniation using anterior approach has yet to be resolved. Some surgeons claim that simple discectomy without fusion is sufficient whereas those who prefer FGI believe that without fusion the chances of recurrence, subluxation, kyphosis and instability increases.^1^,^2^ Some of the surgeons have their own criteria based on their experiences and / or on Evidence Based Medicine but there are still no clear cut decision making criteria on this issue. Hence, we undertook a pre-structured questionnaire based study to analyze the criteria used by 120 neurosurgeons, with at least 12 years surgical experience, on single level cervical disc herniation.

Literature suggests that the most important factors considered before performing FGI were age of the patient,^2^,^4^,^7^,^8^,^10^–^15^ occupation,^2^,^3^,^9^,^10^,^16^,^17^ physical examination findings,^1^,^5^,^6^,^18^–^23^ X-ray findings of cervical spine,^1^,^23^,^24^ MRI findings of cervical spine and any previous operation on same vertebral or disc level.

Our study reveals that age of the patient was the most important criteria for 89% of the participating surgeons as also shown in other studies,^2^,^4^,^7^,^8^,^9^ but sex of the patient had no bearing.^10^,^11^ It seems that before 40 the cervical spine is more flexible and hence during this time the chances of dysfunction and instabilities is high; so with surgical manipulation the risk of instability including disc herniation is higher.^1^,^5^,^6^ After the of age 70, not only are the chances of single level disc herniation very low, but also because of osteophyte formation and decrease in the longitudinal diameter of disc space, cervical spine is in restabilization phase,^5^,^6^,^14^,^18^ therefore the chance of disc herniation is lower even after surgical manipulation. In patients between ages 40-70, most of our participating surgeons believed that other criteria should be considered for FGI such as age, occupation, sign and symptoms and imaging findings.

Patient's occupation was the second important factor that had a bearing on decision making for 87% of surgeons. Heavy jobs can have a negative effect on stability of the cervical spine.^2^,^3^,^9^,^10^,^16^ With recurrent micro-trauma the chances of spine dysfunction and instability is higher. Involvement in heavier occupations also seem to exacerbate the imaging findings of degenerative disc disease on plain X-ray and MRI of the cervical spine.^1^ Many cases of cervical disc herniation occur during heavy manual labor and unusual activity^2^,^3^,^10^,^16^ hence for patients who have undergone a cervical discectomy the risk of delayed instablility is higher. In patients with myelopathy, 66 surgeons believed that it is better to consider FGI but for those with radiculopathy this was not the case. In those patients with spinal cord injury due to a herniated disc the neurological deficit has a negative effect on spine stability.^7^,^8^,^9^,^10^

In the presence of degenerative changes at the interested vertebral or disc level on X-ray of cervical spine, 28 surgeons believed that since the spine is in re-stabilization phase, FGI is not essential, as studies have shown, and that the chance of instability is not significant in such a situation.^23^,^24^ However, in the presence of mild or no degenerative changes at the interested level, 72 neurosurgeons would consider other criteria also as regards to performing FGI.

Presence of signal changes on MRI, such as hypo-hyper signal changes due to gliosis on T1and T2 weighted images, which are detected when myelophaty occur because of chronic compression of cervical disc material and spondylosis on the spinal cord and presence of cystic changes due to myelopathy was an important consideration for 62 surgeons to perform FGI and 48 preferred FGI even in the presence of mild to moderate signal change on vertebral column, such as dehyderation of disc material, degenerative process such as hyperthrophy of facet joint and ligaments of the adjacent level and deformity of the vertebral body seen on T2 and T1. Some surgeons believe that signal changes in the spinal cord, as seen on MRI, suggest not only a greater chance of having spinal instability^1^,^23^,^24^ but also some degree of neurological deficit which can be detected on physical examination. In our study, in the presence of signal change in the spinal cord, 48 surgeons preferred the technique of FGI as with surgical manipulation the chance of developing delayed spinal instability and recurrent disc herniation is still higher in this group of patients. In the survey, 72 surgeons expressed belief that FGI is necessary when previous operations (simple discectomy or discectomy with graft) at the same level had failed. Re-operation had no effect on decision making of 16 neurosurgeons.

Hence, from our study, it is revealed that there is still lack of definitive criteria to suggest the optimal surgical procedure for single level cervical disc herniation and in each patient the surgeon has to make a decision based on his or her experiences and existing literature which itself is still not unidirectional. However, some inferences can be drawn from our study.

FGI can be performed in patients younger than 40 years with heavy jobs, having mylopathy and are already operated same level.
